# Transjugular Intrahepatic Portosystemic Shunt (TIPS) Check and Revision in a Freestanding Outpatient Facility: Safety and Efficacy

**DOI:** 10.7759/cureus.74517

**Published:** 2024-11-26

**Authors:** Stephen P Reis, Steffen Haider, Sidney Brejt, Noor Ahmad, David Sperling

**Affiliations:** 1 Division of Vascular and Interventional Radiology, Columbia University Irving Medical Center, New York, USA; 2 Division of Vascular and Interventional Radiology, NewYork-Presbyterian Hospital, New York, USA; 3 Division of Interventional Radiology, Columbia University Irving Medical Center, New York, USA

**Keywords:** fluoroscopy intervention, office based lab, portal hypertension, transjugular intrahepatic portosystemic shunt (tips), transplant hepatology

## Abstract

Aim

This study aims to evaluate the safety and efficacy of transjugular intrahepatic portosystemic shunt (TIPS) check and revision procedures performed in a freestanding interventional radiology (IR) outpatient facility.

Methodology

A total of 40 patients (male 31:female 9, median age 60 years old) underwent a TIPS check and/or revision at a freestanding IR outpatient facility between 2009 and 2017. Procedures were performed using a mobile C-arm unit under intravenous (IV) moderate sedation, with the patient discharged home on the same day. The decision to perform a TIPS check was based on abnormal surveillance ultrasound findings or the recurrence of portal hypertension symptoms. TIPS were revised if the patient was found to have angiographic stenosis, elevated portosystemic gradients, or recurrent symptoms. TIPS were revised with balloon angioplasty and/or bare metal stent placement.

Results

Revision was attempted in 34 cases, and 6 were found to not require a revision. The average time to revision was 19 months (range 0.6-99 months). Of the 40 patients, 24 (60%) underwent a TIPS check and/or revision due to findings from surveillance ultrasound, while 16 (40%) underwent a check or revision due to a recurrence of symptoms. Of the 34 TIPS revisions, 28 (83%) involved angioplasty alone, 5 (15%) were stent-assisted, and 1 failed due to unsuccessful cannulation. The overall technical success rate for performing the revisions was 94% (32/34). The one-year patency rate was approximately 57%. The mean fluoroscopy time was 16.1 minutes (range: 3.7-52.5 minutes). Post-procedural recovery time was minimal, averaging one to two hours. No major complications were observed (0%, 0/40). One patient (2.5%, 1/40) experienced a minor complication (access site hematoma), and two patients were hospitalized within 30 days for reasons unrelated to the procedure.

Conclusions

TIPS revision can be performed successfully and safely in an outpatient facility.

## Introduction

With the development of alternate practice patterns, office-based interventional radiology (IR) practice, known as the outpatient-based lab (OBL), has gained traction due to its resource efficiency and its ability to provide additional care options and greater convenience for patients. A variety of procedures are now routinely performed in freestanding office-based practices [1-4], but there is a paucity of published data on IR on the safety and efficacy of IR procedures in these venues.

The majority of transjugular intrahepatic portosystemic shunt (TIPS) checks and revisions still occur within hospital settings [5-8]. While TIPS procedures have a high technical and hemodynamic success rate [9-12], they require frequent monitoring for dysfunction. Since the advent of expanded polytetrafluoroethylene (ePTFE)-covered stents, shunt dysfunction has decreased, with one-year patency rates ranging from 79% to 90% [10,12,13]. Despite these improved outcomes, patients with covered stents continue to experience dysfunction, with one recent study showing that approximately half of patients with covered stents will experience dysfunction within two years, including stenosis and hepatic encephalopathy [13]. The treatment of TIPS stenosis includes angioplasty, bare metal stents, or covered stents for in-stent stenosis or for stenosis in the hepatocaval or portal landing zones [5]. Hepatic encephalopathy may be treated by placing a constrained stent to reduce the TIPS diameter.

This study reports our experience with performing transjugular intrahepatic portosystemic shunt checks and revisions in a freestanding outpatient interventional facility and discusses the success and complication rates of these procedures in this setting. The results of this study were previously presented as an E-poster at the 2019 meeting of the Society of Interventional Radiology.

## Materials and methods

The institutional review board approved this retrospective review with a waiver of informed consent. We identified 29 consecutive patients who underwent 40 TIPS checks and/or revisions at a single freestanding IR outpatient facility between 2009 and 2017. Data were collected by retrospective review of the electronic medical record (Allscripts, Chicago, IL) and image review on Picture Archiving and Communication System (PACS, Centricity, GE Healthcare, Chicago, IL).

Indications for initial TIPS creation

The initial TIPS procedures were performed in the hospital setting, with ICU monitoring post-procedure and subsequent inpatient management by the hepatology team. The pre-procedure workup, indications, and contraindications were discussed by a multi-disciplinary team [14]. In this cohort, recurrent variceal bleeding or high-risk gastroesophageal varices were present in 12 patients, refractory ascites in 19 patients, portal vein thrombosis in one patient, and Budd-Chiari syndrome in three patients. One patient underwent TIPS placement to optimize portal hypertension before gynecologic oncologic resection. There were no contraindications to TIPS in this cohort, including, but not limited to, the absence of right heart failure, elevated right heart pressures, or uncontrolled hepatic encephalopathy.

The TIPS procedure was performed using standard techniques, including CO2 cavography for portal vein visualization [15], followed by intracardiac echocardiography (ICE) in the more recent cases [16]. All patients received Viatorr ePTFE-covered covered stent grafts (Gore Medical, Newark, DE).

TIPS follow-up

Following the creation of the TIPS, patients were seen by IR for follow-up one month post-procedure, at which time a hepatic Doppler sonogram was performed. Longitudinal follow-up in the outpatient setting was conducted by our hepatology colleagues. Follow-up TIPS sonograms were then performed every three months on an outpatient basis for up to one year and then every 6 to 12 months thereafter until a sonographic abnormality was detected [5]. Sonographic evidence of TIPS malfunction was defined as TIPS in-stent Doppler velocity greater than 190 cm/second or less than 90 cm/second or a change of over 40 cm/second compared to the prior examination [5,17]. Clinical evidence of TIPS malfunction was defined as the recurrence of ascites, recurrent variceal hemorrhage, edema, or other evidence of recurrent portal hypertension [5,17]. Any case with sonographic and/or clinical evidence of TIPS malfunction not attributable to another cause as determined through discussion between hepatology and IR, was referred for a TIPS cavogram to assess for possible revision.

TIPS check and revision procedures

Patients requiring TIPS downsizing for hepatic encephalopathy were performed on an inpatient basis at the associated hospital. Patients with evidence of TIPS stenosis were eligible for outpatient treatment. Patients underwent a standard pre-procedure workup. TIPS checks and revisions were performed at a freestanding IR facility in a multispecialty office-based facility located approximately 8 miles from the affiliated inpatient hospital. Procedures at this facility were performed using one of two rooms equipped with a Philips Pulsera BV mobile C-arm unit (Philips Medical Systems, The Netherlands), Zonare Ultrasound (Mountain View, CA), and Philips Intellivue MP20 hemodynamic monitors (Philips Medical Systems).

Revision procedures were performed under moderate intravenous sedation and monitored by an appropriately trained registered nurse. Antibiotics were not administered. Procedures were performed by any of the nine interventional radiologists, with experience ranging from two to 30 years. Revision procedures were performed with the right internal jugular access followed by variable sheath sizes to accomplish portal pressure measurements and intervention. If an intervenable lesion was identified by cavogram or an increased hepatic venous pressure gradient (HVPG) was detected, then balloon angioplasty and/or bare metal stents were deployed. Angioplasty cases utilized balloons between 8 and 10 mm in diameter. Four stent cases used balloon-expandable 10 mm diameter bare metal stents, and one case used a self-expanding 12 mm bare metal stent for a portal vein stricture.

Following the procedure, patients were monitored in one of three recovery bays for at least one hour by an appropriately trained registered nurse and then discharged home. The nursing staff followed up with all patients one to two days after the procedure.

Definition of outcomes

This study evaluated the safety and efficacy of TIPS revision in the outpatient setting. The efficacy outcomes were defined as successful access to the TIPS, successful performance of the intervention if necessary, improvement in the pre- to post-procedure gradient, and resolution or improvement of symptoms in symptomatic patients. These factors defined the technical success of the procedure.

Recurrent TIPS malfunction was defined as cavogram evidence of recurrent stenosis or the presence of persistent or recurrent clinical symptoms of portal hypertension. Sonographic evidence of TIPS malfunction, as described above, was always followed by a TIPS check. If a patient did not have hepatic Doppler data at least one-year post-revision, unless a transplant occurred, this was considered a loss to follow-up, even if there were no clinical signs of TIPS malfunction. Endpoints included time to recurrent TIPS malfunction, liver transplant, or death. Recurrent TIPS malfunction was defined as sonographic evidence of stenosis or the recurrence or persistence of symptoms.

All major and minor complications of TIPS revision were classified according to the Society of Interventional Radiology Standards of Practice Committee New Adverse Event Classification [18]. Admission to the inpatient hospital from the outpatient center or within 30 days of revision was also recorded.

Statistical analyses were performed with SPSS Statistics for Microsoft Windows software (version 25.0, IBM Corp., Armonk, NY). Significance was defined as *P* < 0.05. Student's t-test and Fisher's exact test were employed.

## Results

Patient characteristics

Table 1 shows patient characteristics and indications for TIPS checks. Twenty-nine patients underwent 40 TIPS checks and/or revisions at a freestanding IR outpatient facility between 2009 and 2017. The average age of the patients was 58 years, with a male-to-female ratio of 20:9. All these patients initially received Viatorr ePTFE-covered stent grafts (Gore Medical, Newark, DE). The median follow-up was 11 months (range, 0-62 months; standard deviation [SD], 14 months), with 10 patients lacking hepatic Doppler data at least one-year post-TIPS.

**Table 1:** Demographics and indications for TIPS revision. TIPS, transjugular intrahepatic portosystemic shunt

Variable		N or mean (standard deviation or %)
Patients		40
Male:female		31 (77.5%):9 (22.5%)
Age		60 years (10 years)
Time from TIPS creation to revision		19 months (range 0.6-99 months)
Indication for revision		
Surveillance ultrasound findings	24 (60%)	
Increased or decreased velocity	20 (50%)	
Retrograde flow in TIPS	2 (5%	
No flow in TIPS	2 (5%)	
Recurrent symptoms	16/40 (40%)	
Ascites	10/16 (25%)	
Recurrent varices	3 (7.5%)	
Lower extremity edema	1 (2.5)	
Other	2 (5%)	

Timing and indication for follow-up cavogram

The average time from TIPS creation to revision was 19 months (range 0.6-99 months). TIPS checks were performed for surveillance ultrasound findings without new symptoms in 24 cases (60%, 24/40) and for recurrence of symptoms +/- ultrasound findings in 16 cases (40%, 16/40). Ultrasound findings leading to TIPS checks included 20 cases with significant increased or decreased in-stent velocities or lack of flow in the TIPS in two cases each. Of the TIPS checks performed for ultrasound findings alone, 92% (22/24) required intervention.

Intervention and technical outcome

**Figure 1:**
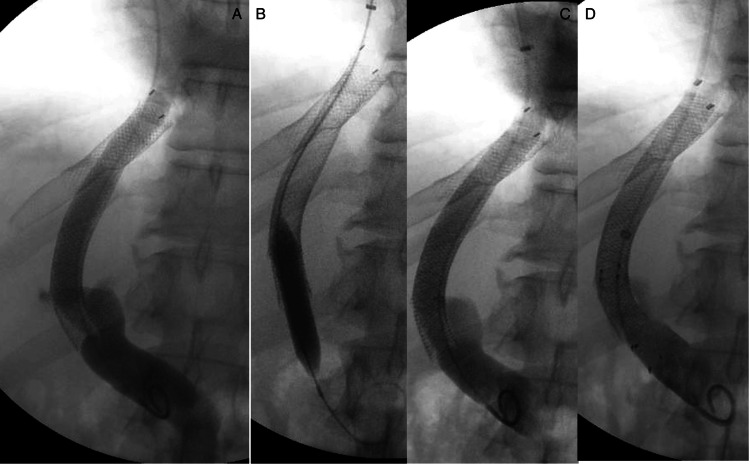
Outpatient TIPS revision: (A) initial cavogram; (B) angioplasty; (C) post-angioplasty; (D) post-stent. A 59-year-old man with alcohol cirrhosis status post TIPS eight years prior, TIPS revision six years prior, and new-onset ascites. TIPS Doppler velocity of 202 cm/second. (A) High-grade stenosis in the inferior stent graft with HVPG 24 mmHg. (B) and (C) Failed 10 mm balloon angioplasty of the stenosis, HVPG 16. (D) 10 mm x 40 mm bare metal stent placement, HVPG 14. Ascites resolved within one month, and the TIPS remained functional in follow-up for five years. TIPS, transjugular intrahepatic portosystemic shunt; HVPG, hepatic venous pressure gradient

Table 2 reports procedural details and technical outcomes. Figure 1 demonstrates an angioplasty and stent-revised TIPS. The intervention was attempted in 34 of the 40 TIPS checks (85%), including 28 cases with angioplasty alone, 5 with bare metal stent placement, and 1 where the TIPS could not be cannulated. In the remaining 6 cases (15%), no intervenable lesion and/or gradient was found and the case was concluded. The overall technical success rate for performing the revision was 94% (32/34). There were two technical failures: the first related to the inability to cannulate the TIPS due to an overlying fibrin sheath and the other due to chronic occlusion of the TIPS despite stent placement. 

**Table 2:** Procedural details and technical outcome. HVPG, hepatic venous pressure gradient; TIPS, transjugular intrahepatic portosystemic shunt

Variable	N or mean (standard deviation, %, range)		
Sedation			
Midazolam		1.3 mg (range 1-2)	
Fentanyl		65 microg (range 25-100)	
Recovery room time	1.2 hours (range 1-2)		
Radiation dose (Air Kerma mGy)	279 mGy (SD 222, 29-1030)		
Fluoroscopy time	16 minutes (SD 11, range 4-53)		
Intervention performed		34 (85%)	
Angioplasty alone		28 (82%)	
Angioplasty and stent		5 (15%)	
Failed cannulation of TIPS		1 (3%)	
No intervention required			6 (15%)
Technical success rate of revisions		32 (94%)	
Unsalvageable TIPS		2 (6%)	
HVPG			
Pre-revision		16 mmHg (SD 7, range 5-31)	
Post-revision		8 mmHg (SD 4, range 0-15)	
Symptomatic outcome		5 cases of symptomatic recurrence or persistent symptoms (13% of 40 cases)	

The baseline mean HVPG before TIPS creation was 20 mmHg (SD 6 mmHg) and post-TIPS creation was 8 mmHg (SD 3 mmHg) (*P *< 0.001). At follow-up TIPS check, the 32 cases in which successful TIPS revision was performed showed a mean HVPG of 16 mmHg (SD 7 mmHg) pre-revision and 8 mmHg (SD 4 mmHg) post-revision (*P* < 0.001 for the increase in gradient from post-TIPS creation to pre-revision, and *P* < 0.001 for the decrease in gradient post-revision). There was no significant change in HVPG post-TIPS creation to TIPS check for the seven cases in which no intervention was attempted. Revision was attempted on seven cases with an angiographic stenosis, but HVPG less than 10 mmHg. Post-TIPS revision, HVPG was 12 mmHg or greater in four cases despite reductions of 10-16 mmHg.

Long-term outcome

Five patients (15.6%) had symptomatic failure of the revised TIPS 2-18 months post-TIPS check and/or revision. Three of these occurred in angioplasty revisions, one each of lower extremity edema, gastric varices, and reaccumulated ascites. One patient post-stent placement had persistent volume symptoms up to liver transplant four months later. One patient with a normal TIPS check developed reaccumulated ascites 12 months later.

**Figure 2:**
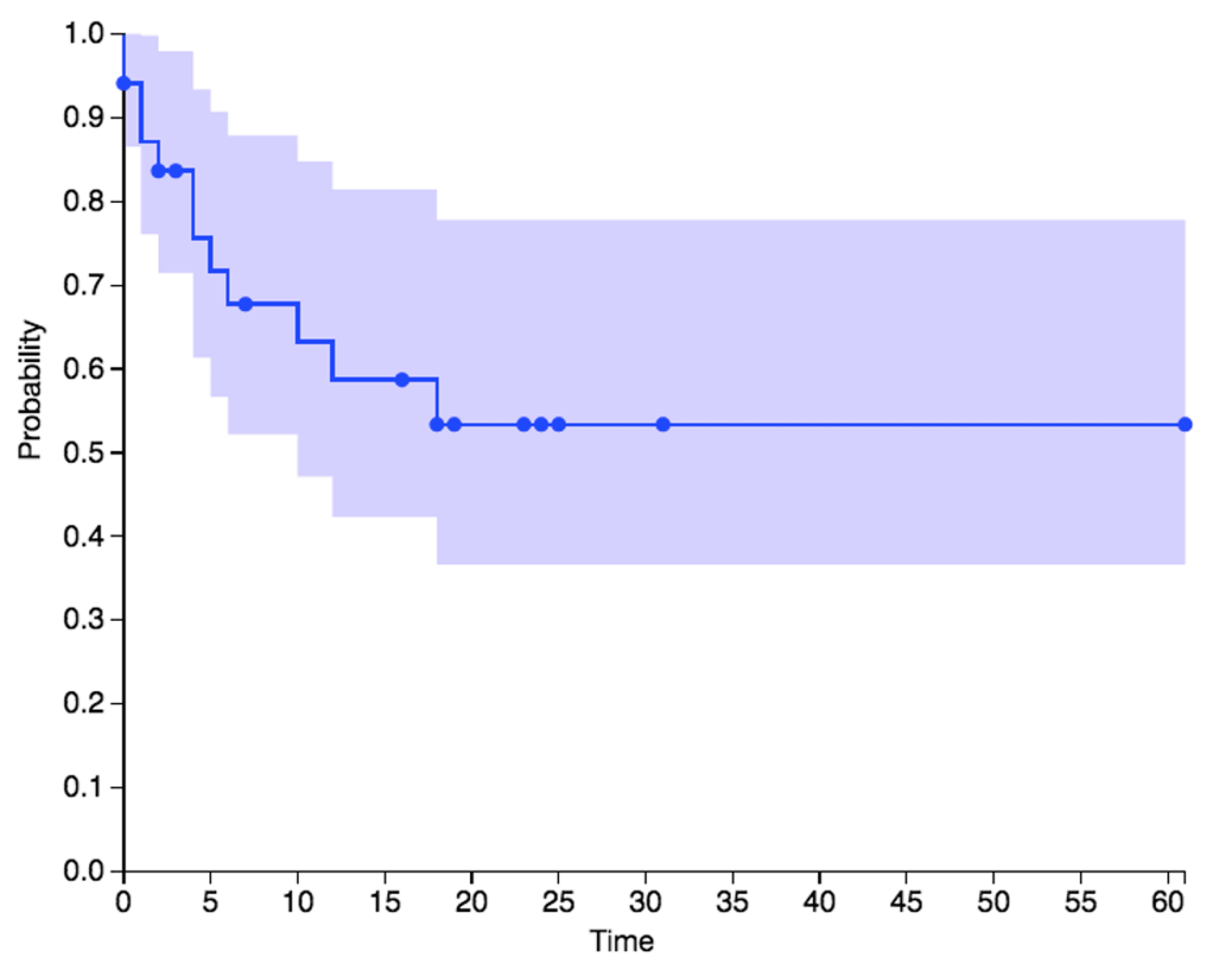
Kaplan-Meier curve of assisted TIPS patency following the 34 revisions in 23 patients. Key: The X-axis is months post-revision. The Y-axis is shunt patency without evidence of dysfunction. Note that this represents 33 TIPS revisions in 23 patients, including 28 angioplasty and 5 bare metal stent placements. Nine patients had two to four revisions each. Note that seven patients were censored before the one-year follow-up without data on TIPS patency. TIPS, transjugular intrahepatic portosystemic shunt

Long-term outcomes were assessed post-revision, as shown in the Kaplan-Meier curve for combined angioplasty and stent-revised cases in Figure 2. Median patency at one-year follow-up was approximately 57% for the combined angioplasty and stent group and 54% for the angioplasty group alone. Following angioplasty revision, there were nine recurrent TIPS malfunctions in five patients, manifested by Doppler velocity changes alone in six patients and both clinical and Doppler recurrence in three patients. Follow-up was limited in the stent group, which consisted of one case with long-term patency, two patients lost to TIPS-specific follow-up, one clinical recurrence, and one technical failure due to chronic occlusion of the TIPS. The patient with four angioplasty revisions demonstrated persistent elevated TIPS velocities on follow-up Dopplers and cavogram, suggesting the poor quality of the underlying TIPS. Among the six cases of TIPS check without revision, two underwent subsequent revision at 4 and 13 months post-TIPS check, respectively.

Radiation dose

The overall average fluoroscopy time was 16 minutes (SD 11 minutes, range 4-53 minutes). The mean radiation dose (Air Kerma) was 279 mGy (SD 222 mGy, range 29-1030 mGy). The largest radiation dose occurred in a patient with failure to recanalize a chronically occluded TIPS; 53 minutes/1030 mGy. Among patients who underwent TIPS revision, the average radiation dose was 297 mGy (SD 218 mGy) with a fluoroscopy time of 16.9 minutes (SD 10.9 minutes). Among patients with only a TIPS check and no attempted revision, the average radiation dose was 171 mGy (SD 239 mGy) with a fluoroscopy time of 8.4 minutes (SD 8.5 minutes).

Sedation and recovery

Conscious sedation with Versed and fentanyl was utilized for patient comfort during the procedure. The average dose of Versed was 1.3 mg (range 1-2 mg) given in 0.5 to 1.0 mg doses. The mean fentanyl dose was 65 microg (range 25-100 microg) given in 25-50 microg doses. The average recovery room time was 1.2 hours (range 1-2 hours). All patients were discharged home from the outpatient office.

Complications

No major complications occurred (0%, 0/40). There were no sedation-related complications. One patient (2.5%, 1/40) had a mild complication (access site hematoma), but the patient was still discharged home. Two patients were admitted to the hospital (5%, 2/40) within 30 days of the procedure, both for unrelated causes. One patient with a TIPS check showing an HVPG gradient of 7 mmHg and no revision had persistent refractory ascites. The patient underwent aggressive inpatient diuresis and TIPS angioplasty two months later, with the HVPG decreasing from 7 to 3 due to mild stenosis, resulting in subsequent volume improvement despite increased hepatic encephalopathy.

## Discussion

There is growing interest in the development of the office-based practice for IR. Our group routinely performs ultrasound-guided biopsies of the liver, kidney, thyroid, and lymph nodes, as well as endovascular procedures such as varicose vein interventions, uterine fibroid embolization, and hepatic transarterial chemoembolization. The safety, time efficiency, and cost efficiency of performing interventional procedures in the OBL setting have been well-established in the vascular surgery literature for peripheral arterial interventions [3,4] and in the nephrology literature for dialysis interventions [1,2]. However, there is a relative paucity of published data on the safety and efficacy of IR procedures performed in freestanding OBLs. This study demonstrated the safety and efficacy of performing TIPS checks and revisions in that setting.

The technical success rate for TIPS revision was 94%, which is comparable to other inpatient studies on the revision of primarily covered TIPS stent grafts [6-8]. Angioplasty alone resulted in assisted TIPS patency of approximately 54% by one year post-intervention, which is comparable to the 50% angioplasty-assisted patency rate reported by Jirkovsky et al. [7]. We note that Jirkovsky et al. also reported one-year assisted patency rates of 88% for dedicated ePTFE-covered stent grafts and 75% for bare metal stents or nondedicated covered stents in the same paper [7]. Furthermore, ePTFE graft revision of primary covered TIPS grafts resulted in assisted one-year patency rates of 100% in Echenagusia et al. [8] and 78%-91% for bare metal stents in a study by Luo et al. [6]. Our group elected to primarily employ angioplasty alone, given that symptomatic recurrence post-TIPS check/revision was only 13% and the group was comfortable following patients clinically and sonographically to assess for recurrent symptoms, necessitating a repeat intervention. This was done to avoid narrowing the TIPS due to the use of covered stents.

While TIPS procedures have a high technical and hemodynamic success rate, they require frequent monitoring for dysfunction. The introduction of e-PTFE-covered stents has significantly reduced rates of shunt dysfunction with recent studies of e-PTFE-covered stents quoting one-year patency rates of around 79%-90% [17,19]. The frequency of surveillance remains institution-specific, with a common algorithm involving hepatic Doppler one month post-TIPS or revision, followed by every three months for the first year, and then every 6-12 months thereafter, until an abnormal Doppler examination occurs [5]. This is the algorithm followed by our group. Eighty-five percent of cases sent for TIPS check were found to require an intervention, suggesting that sonographic and clinical findings are accurate indicators of malfunction. We note that hepatic Doppler may be an insensitive study for TIPS malfunction [20] and indeed clinical symptoms did account for nearly half of the cases. The gold standard for confirming shunt dysfunction remains angiography and cavogram to measure portal pressure gradients [5,17].

Checks and revisions are considered by our group to be safe procedures. A series of TIPS revisions utilizing covered stent-graft placement noted no major complications and 7% minor complications of transient mild hepatic encephalopathy and small access site hematomas [7]. Advanced TIPS recanalization techniques for occluded TIPS have a higher 7% complication rate of bleeding if a trans splenic route is taken [5]. In the present study, there was one minor complication of access site hematoma that did not require admission. There were no major complications. The failed recanalization of the occluded TIPS may have been better served in an inpatient setting in which a transhepatic or trans splenic body floss technique could be employed [17].

Radiation dose utilizing mobile C-arm technology in the outpatient IR suites compared to traditional fixed C-arm technology found in the inpatient setting would be a potential concern. Perhaps counterintuitively, studies have demonstrated higher radiation doses and occupational exposure in the lower extremity and aortic angiography for fixed compared to mobile C-arms, which was attributed to the smaller field of view and lower power of the mobile compared to flat-plate fixed C-arm [21,22]. The choice of mobile C-arm also affects the radiation dose [23], with the dose rate of our Philips C-arms falling between the comparable GE and Siemens models. However, a limitation of this study is that there is no direct comparison in the literature on radiation doses of TIPS revisions. The average radiation dose and fluoroscopy time for this series was 279 mGy and 16 minutes, respectively. This is compared to published data on two hepatic procedures also utilizing digital subtraction angiography: hepatic chemoembolization with 520 mGy and 17.7 minutes and TIP creation with 376 mGy and 7.3 minutes, respectively [24]. The radiation dose rate in the present study compares favorably to these two comparable procedures in keeping with the expected relatively lower radiation dose of mobile C-arm technology at present.

There are several limitations to the present data. This is a single-institution retrospective case series, which increases the chance for systematic bias. This institution is a referral center, and patients may follow up at their home institution, which somewhat limits the availability of follow-up data. Additional limitation includes the somewhat vague definition of TIPS malfunction [5,7]. For instance, seven intervened cases had pre-revision HVPG less than 10 but did have qualitative stenosis on the venogram. Similar to Jirkovsky et al. [7], we had four cases with reduced gradients still 12 mmHg or greater post-revision. Finally, the decision of angioplasty, stent, or no intervention was operator-dependent, introducing variability in treatment into the data.

## Conclusions

The majority of TIPS checks and revisions occur within hospital settings. The purpose of this study is to demonstrate the safety and efficacy of performing this procedure in a freestanding outpatient facility. The ability to perform TIPS revisions on an outpatient basis is more resource efficient, provides patients with more venues and options for care, and increases the convenience and availability of these procedures for patients. This study demonstrates performing transhepatic portosystemic shunt revisions can be performed successfully and safely in an office-based interventional lab.
